# The Utility of Serial Allograft Biopsies during Delayed Graft Function in Renal Transplantation under Current Immunosuppressive Regimens

**DOI:** 10.1155/2014/292305

**Published:** 2014-03-05

**Authors:** Hilana H. Hatoum, Anita Patel, K. K. Venkat

**Affiliations:** ^1^Division of Nephrology, Henry Ford Hospital, 2799 West Grand Boulevard, Detroit, MI 48202, USA; ^2^Division of Nephrology and Transplantation Institute, Henry Ford Hospital, CFP-502, 2799 West Grand Boulevard, Detroit, MI 48202, USA

## Abstract

Delayed graft function (DGF) of kidney transplants increases risk of rejection. We aimed to assess the utility of weekly biopsies during DGF in the setting of currently used immunosuppression and identify variables associated with rejection during DGF. We reviewed all kidney transplants at our institution between January 2008 and December 2011. All patients received rabbit antithymocyte globulin/Thymoglobulin (ATG) or Basiliximab/Simulect induction with maintenance tacrolimus + mycophenolate + corticosteroid therapy. Patients undergoing at least one weekly biopsy during DGF comprised the study group. Eighty-three/420 (19.8%) recipients during this period experienced DGF lasting ≥1 week and underwent weekly biopsies until DGF resolved. Biopsy revealed significant rejection only in 4/83 patients (4.8%) (one Banff 1-A and two Banff 2-A cellular rejections, and one acute humoral rejection). Six other/83 patients (7.2%) had Banff-borderline rejection of uncertain clinical significance. Four variables (ATG versus Basiliximab induction, patient age, panel reactive anti-HLA antibody level at transplantation, and living versus deceased donor transplants) were statistically significantly different between patients with and without rejection, though the clinical significance of these differences is questionable given the low incidence of rejection. *Conclusions*. Under current immunosuppression regimens, rejection during DGF is uncommon and the utility of serial biopsies during DGF is limited.

## 1. Introduction

Delayed graft function (DGF) complicates the early posttransplant period following kidney transplants (KTxs) in approximately 30% of deceased donors and less than 5% of living donor KTx recipients and confers an increased risk of superimposed acute rejection [1–3]. DGF is associated with significantly inferior long-term outcomes in KTx recipients and the outcomes are further worsened when rejection is superimposed on DGF [1–3]. Thus, timely diagnosis and treatment of rejection occurring during DGF are critical. However, noninvasive diagnosis of rejection during DGF is difficult and allograft biopsy is the only reliable test to diagnose it. Therefore, performance of weekly biopsies until DGF resolves is a common practice [4]. Currently, induction therapy using rabbit antithymocyte globulin/Thymoglobulin (ATG) (or, less commonly, Basiliximab/Simulect) is used almost universally during DGF together with potent maintenance immunosuppression (calcineurin-inhibitor + mycophenolate ± corticosteroid). The incidence of rejection during DGF and the continued need for performing periodic biopsies during DGF with currently used immunosuppressive protocols have not been well studied. The aims of our study were to determine the incidence of rejection during DGF and to identify any variable(s) that might predict its occurrence during DGF in the setting of current immunosuppression. We did not aim to study the incidence of DGF or its causes, a subject that has already been studied extensively [1–3], and limited ourselves to assessing the risk of rejection during established DGF.

## 2. Methods 

With Institutional Review Board approval, we reviewed the records of all recipients of KTxs at our institution between January 2008 and December 2011. DGF was defined as a daily decrease in the immediate pretransplant serum creatinine level (SCr) by <10% and/or the requirement of dialysis during the first posttransplant week. Immunosuppression consisted of ATG (1.5 mg/kg daily for 3 days) or Basiliximab (20 mg on days 0 and 4) intravenously started intraoperatively and oral triple maintenance therapy (tacrolimus (target trough level 10 to 15 ng/mL) + mycophenolate (mofetil 1 g or sodium 720 mg Q 12 hr) + methylprednisolone (250 mg intravenously daily—3 doses followed by 30 mg orally daily)) began immediately after transplant. If DGF persisted more than 7 days, weekly allograft biopsy was performed until DGF resolved (defined as daily fall in SCr of ≥20% without dialysis). The patients with DGF were divided into rejection and no-rejection groups based on whether any biopsy done during DGF showed rejection or not. The following variables were recorded in both groups: recipient and donor demographic data, etiology of native renal disease, type and duration of pretransplant dialysis, primary versus retransplantation, number of pretransplant blood transfusions, number of HLA-A, B, and DR mismatches between donor and recipient, immediate pretransplant panel reactive anti-HLA antibody level (PRA), type of donor (living versus deceased), KTx alone versus KTx + another organ, simple cold versus pulsatile pump preservation, cold and warm ischemia times, ATG versus Basiliximab induction, mean tacrolimus trough level, and mean absolute lymphocyte count in the week preceding biopsy. Data are presented as mean ± standard deviation, counts, and/or percentage. Due to the small number of patients with rejection, the two groups were compared using both nonparametric Wilcoxon two-group test and Fisher's exact test; multivariable regression analysis was not performed. Statistical significance was set at *P* < 0.05.

## 3. Results

Out of 420 recipients of KTXs during the study period, 83 patients (19.8%) experienced DGF lasting ≥1 week and underwent weekly biopsies until DGF resolved. The mean age (years) of these 83 patients was 54.5 + 11.7, 65% males, 54% African Americans, 93% deceased donor recipients, and 77% received ATG and 23% Basiliximab induction. Forty-seven patients had one, 30 patients had two, 5 patients had three, and 1 patient had 4 weekly biopsies during DGF. Only 4 of 83 patients (4.8%) had clinically significant rejection on biopsy (one with Banff 1-A acute cellular rejection diagnosed on the second weekly biopsy (treated with 3 daily methylprednisolone intravenous boluses), two with Banff 2-A acute cellular rejection, both diagnosed on the first weekly biopsy (treated with 7 and 10 daily ATG infusions, resp.), and one with acute humoral rejection (treated with 7 daily ATG and 4 daily intravenous immunoglobulin infusions, without plasmapheresis)). All rejections were successfully reversed. Six of 83 patients (7.2%) had Banff-borderline cellular rejection of uncertain clinical significance (though all six were treated with 3 daily methylprednisolone boluses). In the remaining 73 patients biopsy showed only tubular injury of varying degrees with no features of rejection. Although a large number of variables which might have predicted the occurrence of rejection during DGF were examined, only the percentage of patients receiving ATG versus Basiliximab induction, patient age, immediate pretransplant PRA, and the proportion of living versus deceased donor recipients were statistically significantly different between the two groups (Table 1). Importantly, African American recipient race, number of HLA-A/D/DR mismatches, absolute lymphocyte count, and trough tacrolimus levels were not statistically significantly different in the two groups (Table 1).

## 4. Discussion

Our results show that currently used immunosuppression affords good protection against significant rejection even during DGF lasting up to 4 weeks, with only a very small fraction of patients (4.8% in our study) developing this complication. The clinical significance and need for treatment of Banff-borderline rejection that was found in 7.2% of our patients are controversial [5, 6]. Statistically, ATG was more protective than Basiliximab against rejection during DGF. Also, rejection was significantly more common in older patients, those with a lower PRA, and in the group with more living donors, but these differences are counterintuitive because these groups would have been expected to have a lower rejection risk. Thus, the clinical significance of the statistically significant differences we observed is uncertain given the very small number of patients with rejection. Serial biopsies during DGF are a common clinical practice [4]. Biopsy is generally safe, but serious complications including allograft loss and death may occur [7–9]. One study has suggested that, with ATG induction, biopsy to detect rejection during DGF may be unnecessary [10].

Given the low incidence of rejection that we found with current immunosuppression and the successful reversal of all 4 significant rejections (in spite of the fact that biopsies were done only once a week and rejection might have actually set in up to 6 days before biopsy documentation), reduced frequency of biopsies during DGF may be reasonable. We were not able to definitively identify variables that associate with rejection during DGF. If future studies identify such variables, biopsy during DGF can be restricted to patients exhibiting them.

## Figures and Tables

**Table 1:** Comparison of selected variables in the rejection and no-rejection groups.

Variable	Rejection group (*N* = 10; 3 with ≥Banff-1A, 1 with acute humoral, and 6 with Banff-borderline)	No-rejection group (*N* = 73)	*P* value
ATG/Basiliximab induction (%)	**50/50**	**81/19**	**0.044**
Recipient age (years)	58.7 ± 11.0	50.3 ± 12.4	**0.050**
PRA level pretransplant (%)	5.4 ± 17.1	23.0 ± 32.6	**0.021**
Living/deceased donor recipients (%)	**20/80**	**5/95**	**0.010**
Recipient race: African American/other (%)	50/50	55/45	1.000
Number of donor recipient HLA A, B, and DR mismatches	3.9 ± 2.23	4.0 ± 1.63	0.870
Absolute lymphocyte count/mm^3^ in the week preceding biopsy	0.4 ± 0.2	0.6 ± 0.4	0.431
Trough tacrolimus level in the week preceding biopsy (ng/mL)	9.9 ± 2.8	10.6 ± 2.9	0.562
